# Supplementary material for: Reconciling Mining with the Conservation of Cave Biodiversity: A Quantitative Baseline to Help Establish Conservation Priorities
Source: PLoS One. 2016 Dec 20;11(12):e0168348. doi: 10.1371/journal.pone.0168348 (PMC5173368; doi:10.1371/journal.pone.0168348)
Supplement: S1 Dataset — (ZIP) [file pone.0168348.s002.zip › Taxa/Serra Norte/SN_2013/N4WS/Lista N4WS-20.pdf]

CAVIDADE N4WS-0020

Classe	Ordem	Fam/Outros	Gên/Outros	Espécie	S-U	U-S	Zon.
Arachnida	Araneae	Araneidae	*Alpaida*	*septemmammata*	X		E
Arachnida	Araneae	Ctenidae		jovem	3		E
Arachnida	Araneae	Ctenidae		sp.1		1	E
Arachnida	Araneae	Ochyroceratidae		jovem		X	E
Arachnida	Araneae	Pholcidae	*Leptopholcus*	sp.1		X	E
Arachnida	Araneae	Pholcidae	*Mesabolivar*	*eberhard*	X	X	E
Arachnida	Araneae	Scytodidae	*Scytodes*	jovem		1	E
Arachnida	Araneae	Theraphosidae		sp.		1	E
Arachnida	Araneae	Theridiidae	*Achaearanea*	sp.1	X		E
Arachnida	Araneae	Theridiidae	*Episinus*	sp.1		X	E
Arachnida	Araneae	Theridiosomatidae	*Plato*	sp.1	X	X	E
Arachnida	Opiliones	Sclerosomatidae	*Prionostema*	sp.1	X	X	E
Arachnida	Opiliones	Stygnidae	*Protimesius*	*gracilis*	2		E
Arachnida	Pseudoscorpiones	Bochicidae		sp.1	X		E
Arachnida	Pseudoscorpiones	Chernetidae		jovem		X	E
Chilopoda	Scolopendromorpha	Scolopocryptopidae	*Tidops*	sp.1		1	E
Diplopoda	Polydesmida	Fuhrmannodesmidae		sp.1	X		E
Diplopoda	Spirostreptida	Pseudonannolenidae	*Pseudonannolene*	sp.1		1	E
Entognatha	Collembola	Entomobryidae		sp.4	X		E
Entognatha	Collembola	Isotomidae		sp.1	X		E
Entognatha	Collembola	Paronellidae		sp.1	X		E
Entognatha	Diplura	Campodeidae		sp.1	X	X	E
Entognatha	Diplura	Japygidae		sp.1	X		E
Insecta	Archaeognatha	Meinertellidae		sp.1		X	E
Insecta	Blattodea			jovem		3	E
Insecta	Coleoptera	Carabidae		sp.5	X		E
Insecta	Coleoptera	Staphylinidae		sp.66		X	E
Insecta	Coleoptera	Tenebrionidae		jovem		X	E
Insecta	Diptera	Ceratopogonidae		jovem		X	E
Insecta	Diptera	Chironomidae		sp.		X	E
Insecta	Diptera	Ephydridae		sp.		X	E
Insecta	Diptera	Psychodidae	*Evandromyia*	*saulensis*	X		E
Insecta	Diptera	Psychodidae	*Psychodopygus*	Série *chagasi*		X	E
Insecta	Diptera	Psychodidae	*Sciopemyia*	*sordellii*		X	E
Insecta	Diptera	Simuliidae		sp.		X	E
Insecta	Diptera	Tipulidae	Tipulinae	sp.	X		E
Insecta	Heteroptera	Reduviidae	Emesinae	jovem		1	E
Insecta	Hymenoptera	Formicidae	*Camponotus*	sp.1	X	X	E
Insecta	Hymenoptera	Formicidae	*Nylanderia*	sp.3		X	E
Insecta	Hymenoptera	Formicidae	*Pheidole*	sp.2		X	E
Insecta	Isoptera			sp.		X	E
Insecta	Lepidoptera	Noctuoidea		sp.12	11	5	E
Insecta	Lepidoptera	Tineoidea		sp.6		X	E
Insecta	Orthoptera	Phalangopsidae	*Paraclodes*	sp.	9		E
Insecta	Orthoptera	Phalangopsidae	*Phalangopsis*	sp.	22	2	E
Insecta	Psocoptera	Asiopsocidae	*Asiopsocus*	sp.1	X		E
Gastropoda	Pulmonata	Systrophiidae	*Happia*	sp.	X		E
Malacostraca	Isopoda	Philosciidae		sp.2		X	E
Symphyla		Scutigerellidae	*Hanseniella*	sp.1	X		E

Amphibia	Anura	Strabomantidae	*Pristimantis*	cf. *fenestratus*	5		E
